# Prevalence of hereditary tubulointerstitial kidney diseases in the German Chronic Kidney Disease study

**DOI:** 10.1038/s41431-022-01177-9

**Published:** 2022-09-13

**Authors:** Bernt Popp, Arif B. Ekici, Karl X. Knaup, Karen Schneider, Steffen Uebe, Jonghun Park, Vineet Bafna, Heike Meiselbach, Kai-Uwe Eckardt, Mario Schiffer, André Reis, Cornelia Kraus, Michael Wiesener

**Affiliations:** 1 Institute of Human Genetics, University Hospital Erlangen, Friedrich-Alexander-Universität Erlangen-Nürnberg, Erlangen, Germany; 2 Institute of Human Genetics, University of Leipzig Hospitals and Clinics, Leipzig, Germany; 3 Berlin Institute of Health at Charité - Universitätsmedizin Berlin, Center of Functional Genomics, Hessische Straße 4A, 10115 Berlin, Germany; 4 Department of Nephrology and Hypertension, University Hospital Erlangen, Friedrich-Alexander Universität Erlangen-Nürnberg, Erlangen, Germany; 5 Department of Computer Science & Engineering, University of California, San Diego, La Jolla, CA USA; 6 Department of Nephrology and Medical Intensive Care, Charité Universitätsmedizin Berlin, Berlin, Germany

**Keywords:** End-stage renal disease, Genetics research, Alport syndrome, Nephrosclerosis

## Abstract

Hereditary chronic kidney disease (CKD) appears to be more frequent than the clinical perception. Exome sequencing (ES) studies in CKD cohorts could identify pathogenic variants in ~10% of individuals. Tubulointerstitial kidney diseases, showing no typical clinical/histologic finding but tubulointerstitial fibrosis, are particularly difficult to diagnose. We used a targeted panel (29 genes) and *MUC1*-SNaPshot to sequence 271 DNAs, selected in defined disease entities and age cutoffs from 5217 individuals in the German Chronic Kidney Disease cohort. We identified 33 pathogenic variants. Of these 27 (81.8%) were in *COL4A3/4/5*, the largest group being 15 *COL4A5* variants with nine unrelated individuals carrying c.1871G>A, p.(Gly624Asp). We found three cysteine variants in *UMOD*, a novel missense and a novel splice variant in *HNF1B* and the homoplastic *MTTF* variant m.616T>C. Copy-number analysis identified a heterozygous *COL4A5* deletion, and a *HNF1B* duplication/deletion, respectively. Overall, pathogenic variants were present in 12.5% (34/271) and variants of unknown significance in 9.6% (26/271) of selected individuals. Bioinformatic predictions paired with gold standard diagnostics for *MUC1* (SNaPshot) could not identify the typical cytosine duplication (“c.428dupC”) in any individual, implying that ADTKD-*MUC1* is rare. Our study shows that >10% of selected individuals carry disease-causing variants in genes partly associated with tubulointerstitial kidney diseases. *COL4A3/4/5* genes constitute the largest fraction, implying they are regularly overlooked using clinical Alport syndrome criteria and displaying the existence of phenocopies. We identified variants easily missed by some ES pipelines. The clinical filtering criteria applied enriched for an underlying genetic disorder.

## Introduction

Population-based studies imply that genetic kidney diseases are much more frequent than the clinical perception. The complexity amongst hereditary kidney diseases is high, with more than 200 diseases and considerably more candidate genes being associated [1, 2]. A systematic approach using exome sequencing (ES) in a cohort of more than 3.000 individuals with chronic kidney disease (CKD) has recently yielded diagnostic variants in almost 10% of individuals [3]. Further studies with similar results have been published using ES on different individual cohorts, either population based or selected by specific disease entities. In these studies the diagnostic yield has been reported between 7% and 40% depending on population characteristics and selection criteria (e.g., pediatric vs. adult, syndromic vs. isolated, familial vs. simplex) [4]. The number of hereditary kidney diseases is likely higher, since less clear genetic variants and genes not reliably associated with CKD have been excluded and complex genomic regions (such as repeat sequences) and diseases caused by copy number variants (CNVs) may be difficult to identify by ES [5]. Furthermore, mitochondrial diseases are regularly missed since the mitochondrial genome is not targeted in typical ES designs. Therefore, the true prevalence of genetic diseases among individuals with CKD remains ambiguous to date.

A particularly difficult group of hereditary kidney diseases to diagnose are tubulointerstitial kidney diseases. These diseases cannot be recognized by any typical clinical or histopathological signs. They are characterized merely by progressive CKD and secondary features such as hypertension, as well as tubulointerstitial fibrosis in the kidney biopsy. Specific hereditary diseases with a fibrotic, tubulointerstitial phenotype primarily affecting the adult are autosomal dominant tubulointerstitial kidney diseases (ADTKD) [6, 7] and mitochondrially inherited tubulointerstitial kidney diseases (MITKD) [8]. Furthermore, the heterogeneous group of nephronophthisis (NPHP; considered pediatric [9]) would also meet these criteria. Large investigative adult CKD cohorts have shown an unexpected high prevalence of Alport syndrome (AS), affecting the collagen IV α345 molecule [3]. Thus, searching for hereditary diseases with a tubulointerstitial phenotype should reasonably include genes associated with ADTKD, MITKD, NPHP, and AS. Some of these disease entities will not be detected by standard next-generation sequencing techniques, which is particularly true for ADTKD-*MUC1* [10, 11], ADTKD-*HNF1B* where up to 50% of pathogenic variants consist of CNVs [12] and MITKD [8]. Therefore, a comprehensive search for tubulointerstitial diseases should include technological options to detect these diseases.

To investigate the prevalence of these disorders in a large CKD cohort we established a set of clinical criteria to select individuals with increased risk for tubulointerstitial diseases from the >5.000 adult individuals previously recruited into the German Chronic Kidney Disease (GCKD) [13, 14] cohort. In order to ameliorate some of the diagnostic gaps of ES and enable rapid and high-quality sequencing of our cohort, we designed a custom sequencing panel paired with a bioinformatic pipeline enabling analysis of copy number, mitochondrial variants and the *MUC1*-VNTR. Selected samples were subject to sequencing which was supplemented with gold-standard *MUC1*-dupC diagnostics by SNaPshot [11].

Based on the diagnostic yield of our study and in comparison with published screenings, we recommend an algorithm to select individuals with increased risk for a hereditary tubulointerstitial kidney disease for genetic diagnostics and propose sequencing assays and accompanying analysis pipelines for rare kidney diseases.

## Materials and methods

### Ethics and study cohort

This study adheres to the principles set out in the Declaration of Helsinki. The probands included in our study were filtered using the database of the GCKD cohort which enrolled 5217 exclusively Caucasian individuals. The GCKD study is registered as a national clinical study (DRKS 00003971) and was approved by local ethics review boards of all participating institutions [14].

**Fig. 1:**
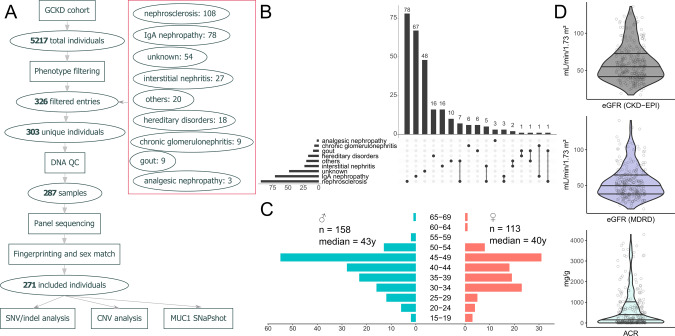
Filtering approach and cohort characteristics. **A** Workflow used to filter individuals from the GCKD and subsequent quality control steps to ensure DNA integrity and sample identity. Clinical category based filtering resulted in 326 entries which corresponded to 303 unique individuals (5.8% of the whole GCKD cohort). Of these 271 (89.4%) passed all quality control steps and were included in the final analyses. **B** Upset plot showing the distribution and overlap of the nine clinical criteria used to filter the study cohort from the GCKD cohort. **C** Age distribution by sex in the final cohort. The y-axis depicts age classes 5-year intervals. The x-axis shows the number of individuals, with females on the right (red) and males on the left (blue) side. Age is reported at inclusion into the GCKD study. **D** Distribution of different kidney function parameters at GCKD study inclusion: Top (dark gray) eGFR by CKD-EPI, Middle (blue) eGFR by MDRD, Bottom (light gray) Albumin-Creatinine Ratio (ACR).

The GCKD database was filtered using nine annotated categories (“nephrosclerosis”, “gout”, “IgA nephropathy”, “chronic glomerulonephritis”, “analgesic nephropathy”, “interstitial nephritis”, “hereditary disorders”, “others”, “unknown”) considering the individual’s age (cutoff ≤50 years, except “IgA nephropathy”, “chronic glomerulonephritis”, “analgesic nephropathy” with ≤40 years and “hereditary disorders” with no age cutoff) as presumed leading CKD etiology. A detailed explanation of the filter criteria is provided in the Supplementary Methods. We excluded all individuals with known postrenal or primary glomerular disease etiology, known systemic disease, known status after acute kidney injury, polycystic kidneys, and those with single kidneys. Biobank DNA samples were subsequently picked and analyzed for quality. All filtering and quality control steps are depicted in Fig. 1A.

### Custom targeted panel design and bioinformatic workup

To design a custom panel covering genes associated with tubulointerstitial kidney disease phenotype the known five genes *MUC1*, *UMOD*, *REN*, *HNF1B*, *SEC61A1* were included [10, 15–18]. To investigate potential bioinformatic approaches of detecting *MUC1* frameshift variants typically located in the VNTR between exon 2 and 3, custom probes covering this region were included and three individuals from families with a *MUC1-dupC* variant confirmed previously by SNaPshot [11] and long-read sequencing [19] were sequenced as controls. We also included three recently published differential diagnoses for ADTKD (genes *DNAJB11*, *GATM*, *PARN*) and 17 nephronophthisis genes. As individuals with ADTKD can have mild to moderate hematuria or proteinuria and therefore could be misdiagnosed, we also included the genes coding for the collagen IV α345 molecule, *COL4A3*, *COL4A4*, and *COL4A5*. Due to the association of tubulointerstitial kidney disease with mitochondrial variants, we added capture probes covering the complete mitochondrial genome. Six gene loci on the X-chromosome (sex computation from coverage) and 24 single nucleotide polymorphism (genomic fingerprinting) markers were added for quality control. Full details on the panel design can be found in File S2.

We developed a custom bioinformatics pipeline to analyze small variants that were defined as “single nucleotide variants” (SNVs) and “small insertions or deletions” (indels), but also copy number variant (CNV) calling from panel data and analysis of the *MUC1*-VNTR region from panel data. A detailed description is provided in the Supplementary Methods.

### Variant evaluation and confirmation

All variants were evaluated for their biological plausibility, examined for quality using the IGV browser, and classified according to the five-tier variant classification system recommended by the American College of Medical Genetics and Genomics (ACMG) [20].

CNVs were validated by orthogonal methods (allele-specific PCR and Sanger sequencing or MLPA). For carriers of a (likely) pathogenic variant in *CEP290*, we performed Sanger sequencing to exclude the deep intronic founder variant NM_025114.3:c.2991+1655A>G. We analyzed the typical cytosine duplication (“dupC”) located at variable positions in the VNTR between exons 2 and 3 of *MUC1* with an established SNaPshot minisequencing protocol for all archived samples selected for panel sequencing. Compare Supplementary Methods for details.

### Comparison with published cohorts, statistical analysis, and plotting

To compare our diagnostic yield and exclude potential biases in variant classification, we compared our analysis to the largest currently published sequencing study in CKD [3]. All data were aggregated into Excel (Microsoft Corporation, Redmond, USA) and analyzed and plotted in R. We used the Wilcoxon signed-rank test, binomial test, or simulation to compute *p* values as appropriate. Compare Supplementary Methods for details.

## Results

### Cohort characteristics

Filtering initially selected 303 individuals from the 5217 individuals of the GCKD cohort (5.8%). 287 (94.7%) DNA samples were of sufficient quality and quantity. Further 16 (5.3%) samples were excluded due to fingerprinting- or sex mismatch, leaving a final cohort of 271 (89.4%) individuals (Fig. 1A). Most individuals fulfilled the inclusion criteria “nephrosclerosis” (94/271 ~ 34.7%), “IgA nephropathy” (71/271 ~ 26.2%) or “unknown” etiology (48/271 ~ 17.7%). 21 individuals (7.7%) were simultaneously in two filtering groups (Fig. 1B). The cohort contained 158 males with a median age of 43 years (range 18–69 years) and 113 females with a median age of 40 years (range 18–66 years), giving a male to female ratio of 1.40 (Fig. 1C) which is comparable with the sex ratio in the whole GCKD cohort (3132/2085 ~ 1.50). Individuals were initially included in the GCKD study following GFR estimation by MDRD study [14] calculation and CKD-EPI equation-based GFR estimates were subsequently performed. We compared these figures for the selected individuals, showing little difference between CKD-EPI (median 55.5 ± 24.2 SD mL/min/1.73 m²) and MDRD (median 50.0 ± 22.0 SD mL/min/1.73 m²) (Fig. 1D middle and top panel). The rate of albuminuria at inclusion in the study is expectedly low as we applied search criteria for tubulointerstitial diseases (median 182.1 ± 886.4 SD mg/g creatinine; Fig. 1D lower panel). The median of the mean per sample read coverage in the 271 final cohort samples is 1929x (min: 384x; max: 4,048). All samples have >99% (min: 99.02%, 173/271 have 100%) of all targeted exons (±20 bp in the introns) covered with ≥20x reads. Compare File S1 [21] sheet for details per individual.

### High diagnostic yield of 12.5% and genetic spectrum

**Fig. 2:**
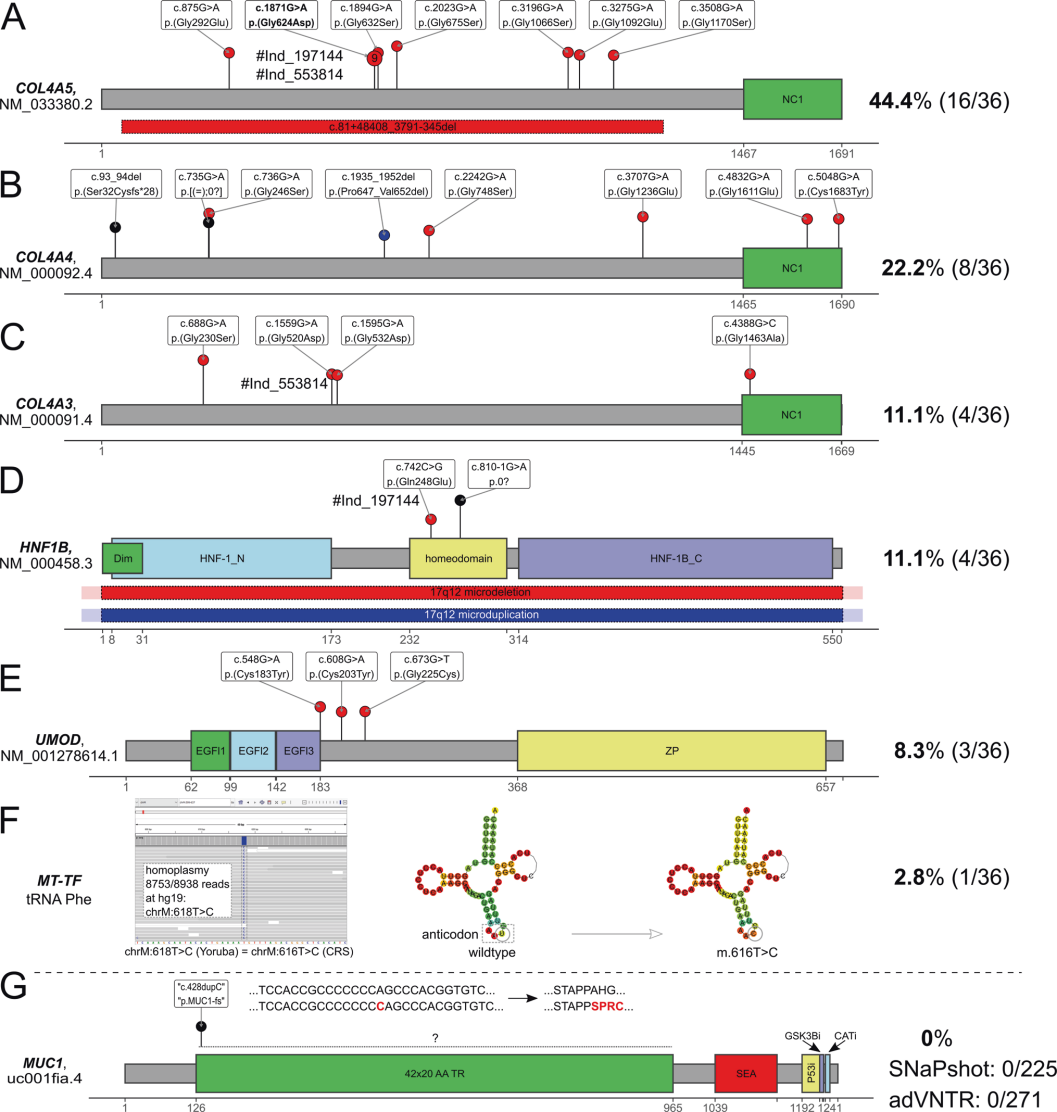
Diagnostic pathogenic variants. Schematic linear protein structure with domains of genes with pathogenic variants identified in the cohort and variant positions marked by lollipops where the length of the segments corresponds to each variant’s CADD score (a computational (“in silico”) metric commonly used to assess the possible pathogenicity of small variants based on an ensemble of annotations like evolutionary conservation). Red dots represent missense variants, black dots represent likely truncating variants, and blue dots represent indels causing in-frame deletions. Red and blue bars with dotted margin represent deletions and duplications, respectively. Individuals with multiple variants identified are linked through the individual pseudonym marked with a “#” under the respective variants. **A** In *COL4A5* we identified 15 SNVs and one intragenic deletion. Note that nine unrelated individuals carried the c.1871G>A, p.(Gly624Asp) variant in either hemizygous (six) or heterozygous (three) states. Two individuals (#Ind_197144, #Ind_553814) carried this recurrent missense and another pathogenic variant. **B** The eight variants identified in *COL4A4* either affected conserved glycine residues directly through a missense change (four), through an in-frame deletion (one) were likely protein truncating variants (two) or affected a cysteine residue in the C-terminal NC-domain. **C** All four variants in *COL4A3* were typical glycine missense changes. One female individual carried the c.1559G>A, p.(Gly520Asp) variant with the recurrent *COL4A5* variant. **D** In four individuals we identified variants affecting *HNF1B*. These were a missense variant in the homeodomain, a splice acceptor variant and a genomic deletion and duplication of the 17q12 region, respectively. Deletion breakpoints could not be determined using the sequencing data or MLPA confirmation (red/blue fill overflowing the margin indicating this uncertainty). One female individual carried the c.742C>G, p.(Gln248Glu) variant with the recurrent *COL4A5* variant. **E** All three pathogenic variants in *UMOD* were typical cysteine missense variants. **F** In the mitochondrial gene *MT-TF*, which encodes the tRNA for phenylalanine, a homoplastic SNV was identified and confirmed. The variant affects the anticodon as predicted through the RNAfold web server [46] and has been listed as pathogenic in MITOMAP [47]. **G** Schematic of the MUC1 protein domain structure and the usually unknown position of the typical cytosine duplication (”c.428dupC”) causing a toxic neo-protein in the VNTR region between exons 2 and 3. Bioinformatic search using adVNTR [48] identified no variant and successful “gold standard” SNaPshot in 228 also identified no positive case in the cohort. Gray dashed line used to separate *MUC1* from genes with diagnostic variants in the cohort. Please compare File S2 [21] sheet “domains” for full information on gene protein domains.

We identified 36 diagnostic variants in six genes (Fig. 2), which could be classified as type 4 (likely pathogenic) or 5 (pathogenic) variants (Table 1) following the ACMG [20] recommendations.

**Table 1:** Diagnostic pathogenic variants.

Gene	Variant	Type	ACMG classification	Individual	Previous Clinical diagnosis	Kidney biopsy	Genetic diagnosis anticipated clinically
*COL4A5*	c.875G>A, p.(Gly292Glu)	SNV	class 4 (*p* = 0.97)	Ind_924166	gout	yes	yes
	c.1871G>A, p.(Gly624Asp)	SNV	class 5 (*p* = 1.00)	Ind_734367	hereditary disorders	no	no
	c.2023G>A, p.(Gly675Ser)	SNV	class 4 (*p* = 0.90)	Ind_408589	hereditary disorders	no	yes
	c.1871G>A, p.(Gly624Asp)	SNV	class 5 (*p* = 1.00)	Ind_553814	hereditary disorders	yes	no
	c.1871G>A, p.(Gly624Asp)	SNV	class 5 (*p* = 1.00)	Ind_674188	hereditary disorders	yes	no
	c.1894G>A, p.(Gly632Ser)	SNV	class 4 (*p* = 0.97)	Ind_523397	hereditary disorders	yes	no
	c.3196G>A, p.(Gly1066Ser)	SNV	class 5 (*p* = 1.00)	Ind_120641	hereditary disorders	yes	no
	c.3275G>A, p.(Gly1092Glu)	SNV	class 4 (*p* = 0.90)	Ind_320658	hereditary disorders	yes	no
	chrX:g.(?_107683936)_(107918029_?)del	CNV	class 5	Ind_739404	hereditary disorders	yes	no
	c.1871G>A, p.(Gly624Asp)	SNV	class 5 (*p* = 1.00)	Ind_276132	IgA nephropathy	yes	no
	c.3508G>A, p.(Gly1170Ser)	SNV	class 5 (*p* = 1.00)	Ind_245000	IgA nephropathy; chronic glomerulonephritis	no	no
	c.1871G>A, p.(Gly624Asp)	SNV	class 5 (*p* = 1.00)	Ind_768032	nephrosclerosis	yes	no
	c.1871G>A, p.(Gly624Asp)	SNV	class 5 (*p* = 1.00)	Ind_197144	nephrosclerosis	yes	no
	c.1871G>A, p.(Gly624Asp)	SNV	class 5 (*p* = 1.00)	Ind_905960	nephrosclerosis	yes	no
	c.1871G>A, p.(Gly624Asp)	SNV	class 5 (*p* = 1.00)	Ind_540052	nephrosclerosis; gout	yes	no
	c.1871G>A, p.(Gly624Asp)	SNV	class 5 (*p* = 1.00)	Ind_902111	unknown	no	no
*COL4A4*	c.5048G>A, p.(Cys1683Tyr)	SNV	class 4 (*p* = 0.90)	Ind_330223	hereditary disorders	no	yes
	c.2242G>A, p.(Gly748Ser)	SNV	class 4 (*p* = 0.90)	Ind_203846	IgA nephropathy	yes	no
	c.4832G>A, p.(Gly1611Glu)	SNV	class 4 (*p* = 0.90)	Ind_641864	IgA nephropathy	yes	no
	c.735G>A, p.[(=);0?]	SNV	class 4 (*p* = 0.90)	Ind_251195	nephrosclerosis	yes	no
	c.3707G>A, p.(Gly1236Glu)	SNV	class 4 (*p* = 0.90)	Ind_591007	nephrosclerosis; interstitial nephritis	yes	no
	c.93_94del, p.(Ser32Cysfs*28)	indel	class 5 (*p* = 1.00)	Ind_805187	unknown	no	no
	c.736G>A, p.(Gly246Ser)	SNV	class 4 (*p* = 0.97)	Ind_218190	unknown	no	no
	c.1935_1952del, p.(Pro647_Val652del)	indel	class 4 (*p* = 0.99)	Ind_712115	unknown	no	no
*COL4A3*	c.688G>A, p.(Gly230Ser)	SNV	class 4 (*p* = 0.90)	Ind_800358	gout	no	no
	c.1595G>A, p.(Gly532Asp)	SNV	class 4 (*p* = 0.97)	Ind_977173	gout; hereditary disorders	no	no
	c.1559G>A, p.(Gly520Asp)	SNV	class 4 (*p* = 0.97)	Ind_553814	hereditary disorders	yes	no
	c.4388G>C, p.(Gly1463Ala)	SNV	class 4 (*p* = 0.90)	Ind_458246	IgA nephropathy	yes	no
*HNF1B*	chr17:g.(?_34914860)_(36105069_?)dup	CNV	class 5	Ind_207310	nephrosclerosis	no	no
	c.742C>G, p.(Gln248Glu)	SNV	class 4 (p = 0.90)	Ind_197144	nephrosclerosis	yes	no
	c.810–1G>A, p.0?	SNV	class 5 (*p* = 1.00)	Ind_861194	others	no	no
	chr17:g.(?_34475214)_(36504124_?)del	CNV	class 5	Ind_958149	unknown	no	no
*UMOD*	c.608G>A, p.(Cys203Tyr)	SNV	class 5 (p = 1.00)	Ind_725568	interstitial nephritis	no	no
	c.673G>T, p.(Gly225Cys)	SNV	class 4 (*p* = 0.90)	Ind_395543	interstitial nephritis	yes	no
	c.548G>A, p.(Cys183Tyr)	SNV	class 4 (*p* = 0.97)	Ind_777983	nephrosclerosis; interstitial nephritis	no	no
*MT-TF*	chrM:g.616T>C	SNV	class 5 (*p* = 0.99)	Ind_151715	unknown	no	no

List of all individuals who had a (likely) pathogenic variant identified, as well as their previous diagnostic group/s and clinical anticipation of the hereditary background and whether they had a renal biopsy. Please compare Supplementary Notes for the calculation of the Bayesian *p* values and File S3 for detailed criteria applied in manual ACMG variant classification.

Note that the calculated posterior *p* values indicate a pathogenic classification for some variants, but this can’t be reached with the current combination rules requiring one strong criteria for class 5.

The main focus of our study was to determine the prevalence of ADTKD in a representative cohort of adult individuals with CKD. Regarding the classical ADTKD associated genes (*MUC1*, *UMOD*, *REN*, *HNF1B*, *SEC61A1*), we found three typical cysteine variants in *UMOD* (NM_001278614.1: c.548G>A, p.(Cys183Tyr); c.608G>A, p.(Cys203Tyr); c.673G>T, p.(Gly225Cys)) and a novel missense (NM_000458.3: c.742C>G, p.(Gln248Glu)), and a novel canonical splice variant (NM_000458.3: c.810–1G>A, p.0?) in *HNF1B*. Copy-number analysis additionally identified a duplication and a deletion of *HNF1B*, respectively, which likely represent larger microdeletions/-duplications. No (likely) pathogenic variants were identified in *REN* and *SEC61A1* or in the non-VNTR region of *MUC1*.

In the targeted mitochondrial genome we identified the homoplastic *MTTF* variant m.616T>C, previously described to cause MITKD [22], in one male individual (“Ind_151715”).

An overwhelming number of variants (28/36 ~ 77.8%) were identified in the *COL4A3*, *COL4A4*, and *COL4A5* gene group. Of the 16 diagnostic variants in *COL4A5*, nine (56.3%) were the previously reported c.1871G>A p.(Gly624Asp) variant (NM_033380.2), which appears to be a relatively frequent founder variant in Europe and has traditionally been described to lead to a milder course of CKD [23, 24], while more recent research [25, 26] suggests a broad spectrum with possible severe phenotypes. According to this, the individuals bearing this variant in our study were dispersed throughout Germany and our kinship calculation indicated no recent relatedness. We additionally identified a 188.5 kilobase large heterozygous *COL4A5* deletion in a female individual for which we were able to determine the exact breakpoints from split reads (chrX:g.107731844_107920385del, NM_000495.4:c.81+48408_3791–345del, p.0).

All 36 (likely) pathogenic variants were identified in 34/271 of the analyzed individuals yielding a diagnostic rate of 12.5%. Interestingly, two (2/34 ~ 5.9%) female individuals with the *COL4A5* variant c.1871G>A, p.(Gly624Asp) showed accompanying diagnostic variants in further genes, *COL4A3* (individual “Ind_553814”) and *HNF1B* (individual “Ind_197144”), respectively. Thus, a dual diagnosis or blended phenotype from two independent disorders can be postulated, and is in line with published numbers for multiple diagnostic loci in rare disease individuals [27] and adult CKD individuals [3]. Reported variants with the respective individual´s clinical criteria are listed in Table 1. Table S1 shows additional variants of unknown significance identified and Table S2 lists the (likely) pathogenic variants identified in nephronophthisis genes.

### Relation of clinical criteria and genetic diagnosis

**Fig. 3:**
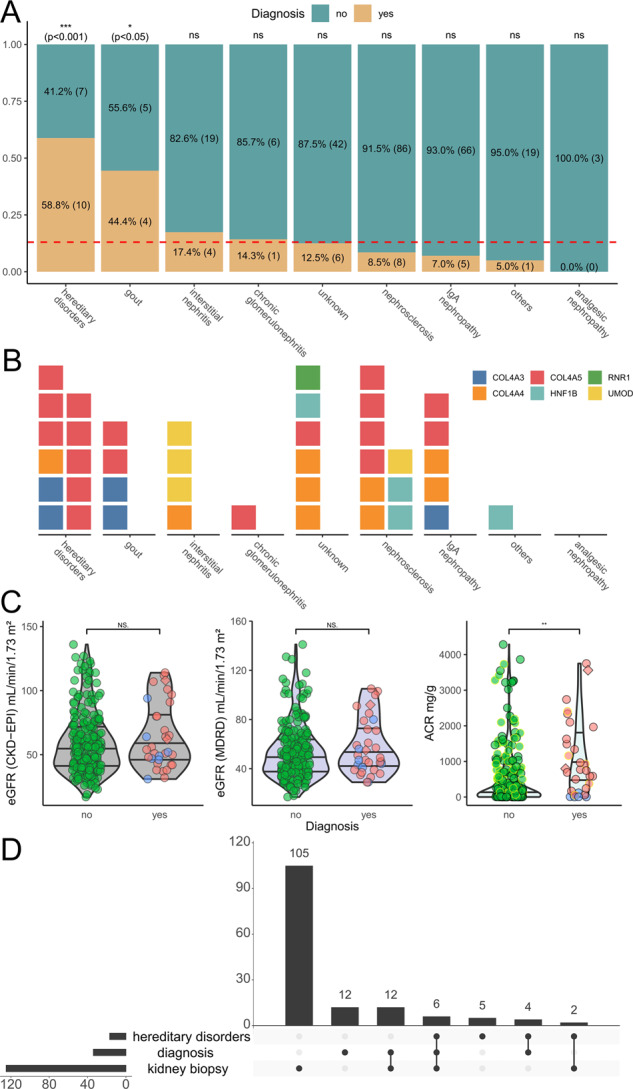
Pathogenic variants by clinical criteria. **A** Stacked bar plot indicating the fraction of individuals with a pathogenic variant identified and split by the nine clinical filtering criteria. As some individuals fulfilled multiple criteria we split them by diagnosis. This resulted in 292 total combinations of individuals and clinical criteria and 39 such combinations for individuals with a genetic diagnosis. To test whether certain criteria are enriched for genetic findings, we calculated *p* values assuming an equal diagnostic rate of 39/292 (red dotted line) in a simple Bernoulli experiment using a binomial test. Categories “hereditary disorders” (*p* ~ 0.000014) and “gout” (*p* ~ 0.023) showed nominally significant enrichment. The “hereditary disorders” category remained significant after adjusting for multiple testing. **B** Waffle plot comparing the nine filtering criteria and the gene in which a variant has been identified. Note that the 39 combinations of individuals and criteria are now also split by gene, because two individuals in the cohort had multiple pathogenic variants, resulting in 41 combinations. The two significant categories from A are all explained through variants in the *COL4A3*, *COL4A4*, and *COL4A5* genes. Interestingly all three *UMOD* variants identified fall in the “interstitial nephritis” category, with one of them additionally classified as “nephrosclerosis”. Variants affecting *HNF1B* are either dispersed through four categories with none of them in the hereditary category, confirming both the variability in the *HNF1B*-associated disorders and the often sporadic nature of the CNVs (17q12 microdeletion/-duplication syndromes). Compare File S3 [21] for full variant details. **C** Violin and scatter plots comparing the kidney function parameters from Fig. 1D between individuals with a genetic variant identified (34) or not (237; green circles). Individuals with a *COL4A3*, *COL4A4*, or *COL4A5* variant are presented in red and with variants in other genes in blue. Individuals with two variants are marked as diamonds. Individuals with IgA nephropathy are marked with yellow margin (compare also Fig. S2). The ACR at GCKD study inclusion is significantly higher in individuals with a genetic variant identified (two-sided Wilcoxon signed-rank test). **D** Upset plot showing the overlaps for individuals with a suspected “hereditary diagnosis” (as used for filtering), our finding of a pathogenic variant (“diagnosis”) and kidney biopsies performed. Overall in only six individuals with a confirmed diagnosis a kidney biopsy had been performed previously which likely raised the suspicion of an underlying genetic disorder. In 12 individuals with kidney biopsy where we identified pathogenic variants no suspicion of a hereditary disease was issued.

Having identified the individuals with an underlying genetic disease, we re-analyzed and correlated the clinical information that was available in the GCKD database. As could be expected, the group “hereditary disorders” harbored the highest rate of individuals with diagnostic variants (10/17 ~ 58.8%), followed by the groups “gout” (4/9 ~ 44.4%) and “interstitial nephritis” (4/23 ~ 17.4%) (Fig. 3A). The large groups of “nephrosclerosis” and “IgA nephropathy” display lower rates of diagnostic hits with 8.4% (8/94) and 7.0% (5/71), respectively. Assuming an equal diagnostic rate for all categories as null hypothesis, only the categories “hereditary disorders” (*p* ~ 0.000014; binomial test) and “gout” (*p* ~ 0.023; binomial test) showed significant enrichment for genetic findings. The group “hereditary disorders” would remain significant when correcting for multiple testing at a threshold of 0.005/9 (~0.0056). By far the most diagnostic variants involve one of the three *COL4A3/4/5*-genes, which are the sole variants in the groups “gout”, “hereditary disorders” and “IgA nephropathy” (Fig. 3B). The diagnostic groups “interstitial nephritis”, “nephrosclerosis” and “unknown” show a higher rate of genetic heterogeneity. However, the numbers are too small to speculate about a systematic effect.

Comparison of the GFR at inclusion into the GCKD study between the group of individuals where a genetic diagnosis was identified with the rest of individuals did not show a difference (Fig. 3C, left and middle panel). In contrast, the albuminuria at inclusion in the study was significantly higher in the genetically determined group, which is an effect exclusively caused by the individuals with AS (Fig. 3C, right-hand panel).

Next, we were interested in the contribution of previous kidney biopsies for the clinical evaluation, since the kidney histology is not informative for the diagnosis of ADTKD [6, 7], but in contrast, could be helpful in recognition of AS. Figure 3D shows that a biopsy was taken in 46.1% (125/271) of the selected individuals before inclusion into the GCKD study. Interestingly, this rate was similar with 47.1% (8/17) in individuals that were classed into the group of “hereditary disorders” and with 52.9% (18/34) in the group of individuals with diagnostic variants. Considering only the individuals with an identified variant in the *COL4A3, COL4A4, or COL4A5* gene, 63.0% (17/27) of this group were biopsied, but only three individuals (11.1%) were previously marked with a suspected diagnosis of AS in the GCKD files (two biopsied, one not biopsied). All these three individuals were correctly positioned by the nephrologists into the group “hereditary disease”, where the rest of individuals in this disease group were commented as “unspecified”. Therefore, for the individuals analyzed in our study, the kidney biopsy does not appear to have been of any direct diagnostic value, unless for exclusion of another disease.

### Comparison with published CKD cohorts confirms high diagnostic rate

**Fig. 4:**
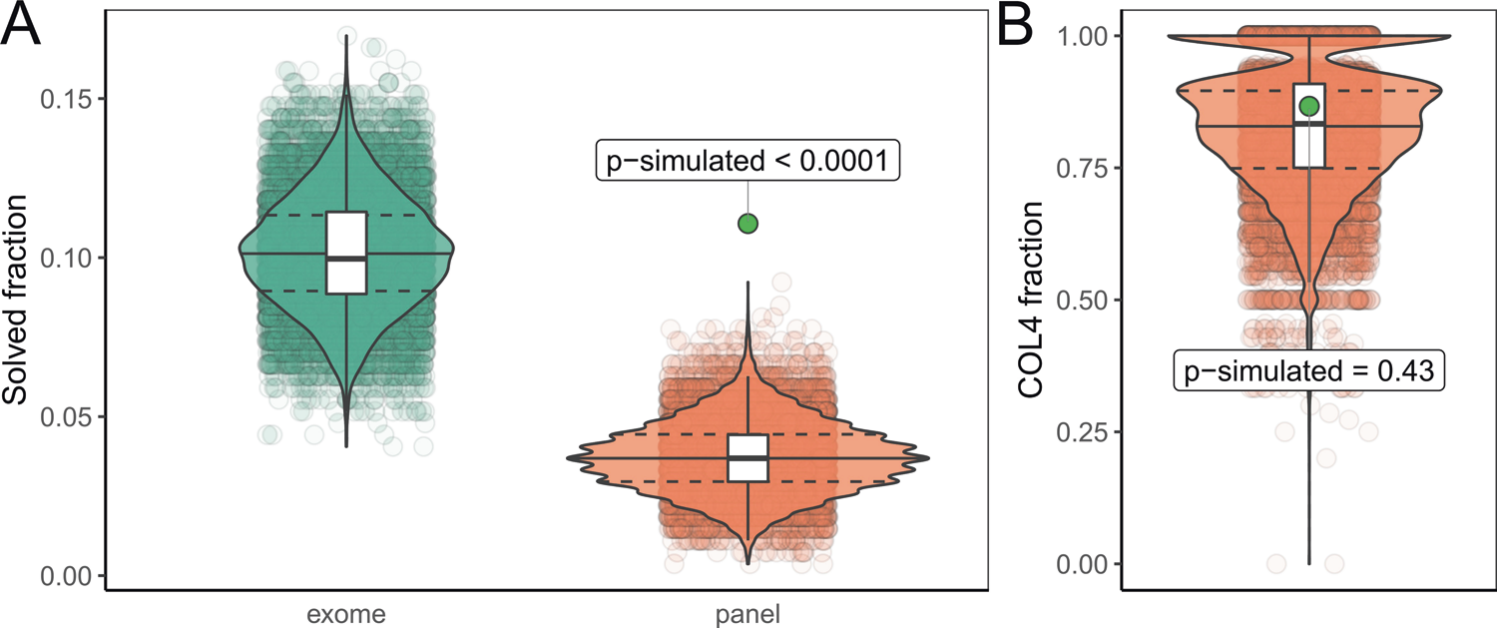
Diagnostic yield in panel and exome. **A** Violin and scatter plots of 10.000 simulations randomly drawing 271 individuals from the 3315 individuals reported by Groopman et al. and subset whether the reported variants would be detectable by exome (green) or our targeted panel (red). Estimated *p* value for the yield in our cohort (green dot) <0.0001. To exclude differences in variant classification between the two studies, we classified both our and all variants from the Groopman study using two automated ACMG classifiers which excluded all variants not classified as (likely) pathogenic from both cohorts. This gave similar results to the first simulation and thus excluded systematic differences in manual variant classification causing our higher yield (compare Figure S1). **B** To exclude unexpected enrichment for *COL4A3*, *COL4A4*, and *COL4A5* genes, we further compared the fraction of variants in these genes in these simulations which showed no significant difference (p-simulated ~0.43) to the fraction (26/30 ~ 86.7%) observed in our cohort. Therefore, one could consider the *COL4A3/4/5* variants as background, which would leave five small variants in ADTKD genes in our cohort, representing an enrichment of ~5.1 fold when compared to the Groopman cohort (calculation: (5/271)/(12/3315)). Compare Figure S1 for automated classification results. Compare File S4 [21] for full simulation results.

Compared to previous studies of adult CKD cohorts, our diagnostic yield of 12.5% (34/271) is relatively high and comparable to exome sequencing, despite the relatively small number of genes in our design and exclusion of *PKD1*/*2* associated disease. To test for the generalizability of this observation, we compared our diagnostic yield to the currently largest exome sequencing study in adults with CKD by Groopman et al. [3]. As this study did not analyze CNVs and mitochondrial variants, we also only included small variants in the autosomes and gonosomes from our study (30/271 ~ 11.1%; excluding CNVs and mitochondrial variants) for the comparison. After harmonizing both our and the AURORA and CUMC cohorts [3] using the same annotations, we performed a simulation where we randomly selected 271 individuals from the 3315 individuals reported with diagnostic variants by Groopman et al. and then counted whether the respective variant reported would be detectable by our analysis. The simulation indicated that our diagnostic yield of 11.1% is very unlikely by chance (estimated *p* value < 0.0001) (Fig. 4A), and this indicates enrichment through our filtering (compare Fig. 4B).

## Discussion

CKD is a frequent disease, affecting more than 10% of the global population, that is strongly associated with adverse prognosis and has a profound socioeconomic impact [28, 29]. In the last decade genetic diagnostics have greatly improved, which has led to the recognition of a relevant burden of hereditary causes amongst individuals with CKD. In parallel, international initiatives promote targeted treatment developments for rare diseases. The aim for “precision medicine” therefore thrives for an accurate diagnosis and an effective targeted therapy [1, 2, 30].

ES on a clinical basis in every individual with CKD is not (yet) realistic, is not standardized (e.g., different commercial designs and bioinformatic pipelines) and has diagnostic gaps for several kidney disorders. Thus, algorithms need to be defined to decide which individual should be offered genetic testing and which combinations of ES and specialized targeted analyses will result in highest diagnostic yields while being as economical as possible for the healthcare system, also taking analysis time into consideration. Increasingly, an ES-based sequencing platform with initial phenotype-oriented virtual panel analysis followed by stepwise expansion of the analysis if the targeted analysis was uninformative is propagated [31]. Possible criteria to undertake genetic analysis would be young CKD onset, disease type, and positive family history as well as the existence of extrarenal, syndromic features [4]. Importantly, the genetic heterogeneity of distinct kidney disease subtypes may also influence the diagnostic sensitivity and influence the choice of sequencing methods [3, 4]. To date, the largest genetic study published on CKD individuals analyzed a virtual panel of 625 genes associated with kidney disease on an exome platform [3]. In this study, 63% of diagnostic variants were restricted to six genes (*PKD1*/*2*, *COL4A3*/*4*/*5*, *UMOD*). Therefore, on a clinical basis, it appears appropriate to restrict the number of analyzed genes. We here used a panel of merely 29 genes to investigate the prevalence of hereditary tubulointerstitial diseases. Our rate of diagnostic findings was higher compared to Groopman et al. (12.5% vs. 9.3% or 10.1 when including their “putatively diagnostic variants”) [3], which we interpret as confirmation of successful filtering criteria (Fig. 4).

The majority of our diagnostic findings were amongst the collagen IV α345 molecule (Fig. 2), which would not normally account for tubulointerstitial but glomerular diseases. However, AS has been extensively reported as frequent unexpected diagnoses in individuals with focal segmental glomerulosclerosis upon renal histology [32], individuals with simultaneous diagnosis of IgA-nephropathy [33] or broad population-based analyses [3, 34], where previous erroneous diagnoses may have taken place. By clinical similarities or atypical clinical courses, phenocopies of the AS and other glomerular diseases may be caused [3, 35]; in single patients this may even mimic tubulointerstitial diseases [36]. Therefore, we decided to include the *COL4A3, COL4A4*, *and COL4A5* genes. The rate of variants in these genes may be lower in other populations since about one-third of the pathogenic variants we found were the *COL4A5* hotspot variant c.1871G>A (p.Gly624Asp) (10/28 ~ 35.7% here vs. in the Groopman study 9/108 ~ 8.3%), with a high frequency in central European populations [23, 24]. Interestingly, looking back into the original entries of the GCKD database, of the 28 individuals with a diagnostic *COL4A3/4/5* variant, only three were previously diagnosed to have AS. Thus, the majority of almost 90% of AS were clinically not recognized. Analysis of the here defined AS individuals for proteinuria showed a significant difference for a moderate proteinuria as compared to the group without a genetic diagnosis (Fig. 3C). Therefore, recognition of proteinuria could sensitize nephrologists towards AS and encourage a restricted diagnostic workup. Overall, our analysis confirmed previous studies showing a high background rate of *COL4A3*, *COL4A4*, and *COL4A5* variants in CKD cohorts. Considering that two individuals with a pathogenic *COL4A3* or *COL4A/5* variant had a second pathogenic variant (2/34 ~ 5.9%) and thus a dual diagnosis, it seems sensible to perform a broader search in individuals with a *COL4A3, COL4A4, or COL4A5* variant, especially if the affected person shows an atypical disease course or additional features.

Further rather difficult diagnostic groups, prone to faulty classification could be “nephrosclerosis” and “IgA nephropathy”, where our analysis yielded diagnostic variants in 8.5% and 7.0% of individuals, respectively (Fig. 2A). Although these groups showed no statistically significant enrichment, when compared to the baseline diagnostic yield in our cohort, they could motivate clinicians to look more careful at individuals before diagnostic classification. Naturally, the great majority of CKD individuals will show arterial hypertension and often it will not be clear if this is the cause or sequel of CKD. Similar challenges can be met with the histological diagnosis of “IgA nephropathy”, which can be found in a substantial fraction of the (healthy) population [37–39]. Therefore, parallel and possibly more severe diagnoses such as ADTKD can be overlooked [40].

Our study investigated a diagnostically particularly difficult group of individuals with hereditary tubulointerstitial diseases. Since individuals suffering from ADTKD usually reach ESRD between the 3rd and 6th decade of life [6, 7] and the GCKD inclusion criteria was CKD stage 3 [14], we set the age cut-off for most leading diagnoses to 40 or 50 years of age (see Methods and Supplementary Methods). This stringent age-related cut-off accepts that single individuals may be missed with an exceptionally mild phenotype, which however is rarely the case with ADTKD [41, 42]. The comprehensive diagnostic difficulties are clinical and histological but also methodological in terms of molecular genetics. As such, respective candidate genes may have frequent CNVs (i.e., *HNF1B*), are not contained in usual genetic screens (mitochondrial genome) or show complex repeat structures (i.e., *MUC1*). In the absence of family history it is very difficult to raise a clinical suspicion of these diseases. Therefore, we suspect that individuals with sporadic disease will hardly be recognized. Thus, the prevalence of these diseases is not known to date. We identified seven variants in known genes for ADTKD which represent 2,6% (7/271) of the sequenced cohort. Interpolating to the total GCKD cohort, while assuming complete enrichment through our criteria, this would mean a prevalence of 0.13% (7/5,217). Interestingly, this figure is similar to another recent study with the estimate of 0.54% for individuals with ADTKD in the complete ESRD cohort of Ireland [43] and the diagnostic yield for ADTKD variants reported by Groopman et al. with 0.39% (13/3315). Importantly, none of the seven individuals with diagnostic ADTKD variants were originally grouped as suffering from a “hereditary disorder”. These individuals were originally classed as “nephrosclerosis” (3x), “interstitial nephritis” (3x), “others” (1x), and “unknown” (1x) (see Table 1). Therefore, we presume that the majority of these diseases could be sporadic.

While we present a detailed analysis of the prevalence of hereditary tubulointerstitial kidney diseases, it is important to consider confounders. First, any selection strategy has the potential to miss individuals. Thus, the true figure of hereditary disease will presumably be higher than our results. Second, we performed a screening limited by a customized gene panel which can miss other causative variants. However, the focus of our study was ADTKD and related diseases, which were tested exhaustively. Third, we classed the variants following the recommendation of the ACMG [20], where class 3 VUS are not contained in our yield calculations. We performed a detailed analysis of these VUS (Table S1). However, it currently remains unknown how many of them in fact are the reason for CKD in single individuals and further population and functional studies (e.g., saturation mutagenesis) will be needed to elucidate their effects. Fourth, we did not include the genes recommended to be reported as secondary findings [44], which are expected in ~1% of the population and are of clinical relevance especially for CKD individuals with chronic dialysis or immunosuppression [4].

In summary, ADTKD/MITKD are quite rare in the CKD population. With limitations in financial resources, it is probably not justified to broadly perform targeted ADTKD diagnostics in the clinical routine, at least in sporadic cases. This is particularly true for ADTKD-*MUC1*, where testing for the “dupC”-variant using SNaPshot is laborious and did not lead to a single hit here. On the other hand, our bioinformatic assessment of the targeted VNTR region showed complete agreement. Also, when clinical criteria are present and a clear autosomal dominant pedigree is evident, the rate of diagnostic variants for ADTKD is reasonably high [41, 45]. Based on these considerations, our and others’ results and experience from rare disease studies we recommend a clinically enhanced ES design paired with customized bioinformatics (Fig. S3A) and an iteration of genetic diagnostics and research re-evaluation (Fig. S3B). Only by establishing such comprehensive workflows in centers for rare kidney diseases will we be able to improve diagnostics, gather further knowledge on each genetic CKD entity and finally improve outcomes.

## Supplementary information

Supplementary Notes

## Data Availability

All data generated or analyzed during this study can be found either in the online version of this article at the publisher’s website or has been uploaded to Zenodo (File S1, S2, S3, S4: 10.5281/zenodo.5516388).
