# Supplementary material for: Prevalence of hereditary tubulointerstitial kidney diseases in the German Chronic Kidney Disease study
Source: Eur J Hum Genet. 2022 Sep 13;30(12):1413–22. doi: 10.1038/s41431-022-01177-9 (PMC9712573; doi:10.1038/s41431-022-01177-9)
Supplement: Supplementary file 1 — Supplementary Notes [file 41431_2022_1177_MOESM1_ESM.pdf]

SUPPLEMENTARY NOTES FOR:

Prevalence of hereditary tubulointerstitial kidney diseases in the German Chronic Kidney Disease study

Bernt Popp[1,2,3,*], Arif B. Ekici[1,*], Karl X. Knaup[4], Karen Schneider[4], Steffen Uebe[1], Jonghun Park[5], Vineet Bafna[5], Heike Meiselbach[4], Kai-Uwe Eckardt[4,6], Mario Schiffer[4], André Reis[1], Cornelia Kraus[1], Michael Wiesener[4]

[1]Institute of Human Genetics, University Hospital Erlangen, Friedrich-Alexander University Erlangen-Nürnberg, Erlangen, Germany
[2]Institute of Human Genetics, University of Leipzig Hospitals and Clinics, Leipzig, Germany
[3]Berlin Institute of Health at Charité - Universitätsmedizin Berlin, Center of Functional Genomics, Hessische Straße 4A, 10115 Berlin, Germany
[4]Department of Nephrology and Hypertension, University Hospital Erlangen, Friedrich-Alexander Universität Erlangen-Nürnberg, Erlangen, Germany
[5]Department of Computer Science & Engineering, University of California, San Diego, La Jolla, CA, USA
[6]Department of Nephrology and Medical Intensive Care, Charité Universitätsmedizin Berlin, Berlin, Germany

[*]both authors contributed equally

CORRESPONDING AUTHOR:
Prof. Dr. Michael S. Wiesener, M.D.
Department of Nephrology and Hypertension
University Hospital Erlangen
Friedrich-Alexander-University Erlangen-Nürnberg
Ulmenweg 18
91054 Erlangen, Germany
Tel: +49 9131 85 39002
Fax: +49 9131 85 39209
Email: michael.wiesener@uk-erlangen.de

Extended acknowledgements

Current GCKD investigators and collaborators with the GCKD study are: University of Erlangen-Nürnberg: Kai-Uwe Eckardt, Heike Meiselbach, Markus P. Schneider, Mario Schiffer, Hans-Ulrich Prokosch, Barbara Bärthlein, Andreas Beck, André Reis, Arif B. Ekici, Susanne Becker, Ulrike Alberth-Schmidt, Anke Weigel; University of Freiburg: Gerd Walz, Anna Köttgen, Ulla T. Schultheiß, Fruzsina Kotsis, Simone Meder, Erna Mitsch, Ursula Reinhard; RWTH Aachen University: Jürgen Floege, Turgay Saritas; Charité, University Medicine Berlin: Elke Schaeffner, Seema Baid-Agrawal, Kerstin Theisen; Hannover Medical School: Hermann Haller; University of Heidelberg: Martin Zeier, Claudia Sommerer, Johanna Theilinger; University of Jena: Gunter Wolf, Martin Busch, Rainer Paul; Ludwig-Maximilians University of München: Thomas Sitter; University of Würzburg: Christoph Wanner, Vera Krane, Antje Börner-Klein, Britta Bauer; Medical University of Innsbruck, Division of Genetic Epidemiology: Florian Kronenberg, Julia Raschenberger, Barbara Kollerits, Lukas Forer, Sebastian Schönherr, Hansi Weissensteiner; University of Regensburg, Institute of Functional Genomics: Peter Oefner, Wolfram Gronwald, and Department of Medical Biometry, Informatics and Epidemiology (IMBIE), University Hospital of Bonn: Matthias Schmid, Jennifer Nadal.

SUPPLEMENTARY METHODS

Detailed explanation of filter criteria selection

The GCKD cohort consists of more than 5.000 adult individuals with chronic kidney disease of stage 3 or overt proteinuria[1,2] at entry into the registry (eGFR 30 – 60 ml/min). If financial resources were unlimited, it would be preferable to analyse all CKD individuals by whole exome sequencing. However, it appears feasible to detect most individuals with a hereditary cause of CKD by selecting the most likely probands by distinct criteria and restrict the molecular analysis to the most likely genes.

All participating nephrologists were asked to enter the leading causative reason for CKD, where competing diagnoses were possible. In general, we focused on the diseases which have the potential of being partly or totally misclassified. Usually, hereditary diseases such as ADTKD lead to end stage renal disease (ESRD) latest by 60 years of age. Since most diseases of interest develop over many years if not decades we hypothesize that most individuals will have reached CKD stage 3 latest by the age of 50. Therefore, for most categories we have filtered for individuals equal or below 50 years of age. The single exception of this rule is the category of "hereditary disease", where the clinicians will have recognized a positive family history of CKD and thus we did not implement an age restriction. For this group the database provides the defined diagnosis for many distinct diseases, which were excluded for further analysis by individual calling.

individuals with certain diseases where the diagnosis is usually an accurate call, we excluded completely from further analysis. Amongst these were diseases such as ADPKD, microscopic polyangiitis, aHUS/TTP, membranous nephropathy or Lupus erythematodes . We did not exclude individuals where diabetes mellitus was entered as the leading cause, since additional development of diabetes is not so unlikely in any individual with a hereditary kidney disease, considering its incidence in the normal population.

The group of single kidneys consisted of more than 85% of cases with surgical removal of one kidney (i.e. tumors, live donation, trauma). Since this does not correspond to ADTKD medical history (in particular not HNF1B-related), we decided to exclude this group also.

Certain diagnoses are in our experience regularly prone to inaccuracy for different reasons. These diagnoses are analgesic nephropathy, chronic glomerulonephritis and IgA nephropathy. In the latter the pathologist may be tempted to discuss IgA, when he sees chronic degenerative histopathology with some amounts of mesangial deposition of IgA. This may in some cases be false, or not the dominant reason for development of CKD. However, since most of these calls will be correct, in these cases we decided to imply more stringency and reduced the age cutoff to 40 years of age, or lower.

Bioinformatic pipeline

Resulting sequence files in BCL format were demultiplexed using bcl2fastq version v1.8.4 (Illumina, Inc., San Diego, CA, USA). Resulting paired reads in FASTQ format were aligned to the hg19 reference genome using BWA-MEM [3] version 0.7.14-r1136. PCR duplicate reads were removed with Picard tools (http://broadinstitute.github.io/picard/) version 1.111 and local realignment of indels was performed using Genome Analysis Toolkit (GATK) [4] version 3.8-0 to produce final BAM files (for alignment statistics see File S1 sheet “BAM_files”).

Small variants were defined as “single nucleotide variants” (SNVs) and “small insertions or deletions” (indels) and were called from the final BAM files using GATK HaplotyeCaller [5] version 4.1.4.0 in genomic variant call format (gVCF) mode. The resulting gVCF files were jointly genotyped with the GATK commands “GenomicsDBImport” and “GenotypeGVCFs” to produce one multi-sample VCF for the whole sequenced cohort. To calibrate and normalize, we split the cohort VCF by variant type using the “SelectVariants” command from GATK, applied recommended hard filtering to both variant sets using the “VariantFiltration” command, merged the filtered VCFs using “MergeVcfs” command and finally normalized and split multiallelic sites using the “LeftAlignAndTrimVariants” command to produce the final VCF.

SnpEff[6] and SnpSift[7] were used to annotate the resulting cohort VCF with variant consequences and information from dbNSFP[8] version 4.0a. Additionally, we annotated splice prediction scores from SPIDEX/SPANR[9] version 1.0 and from dbscSNV[10] version 1.1 and clinical variant assessments from the ClinVar [11] database (status 2020-02-10) and from HGMD [12] version 2019.3.

The annotated variants were filtered to pass calibration, have an allele frequency < 5% in the cohort and < 1% in gnomAD exomes/ genomes with no homozygotes allowed, not being annotated as (likely) benign, while keeping all variants annotated as (likely) pathogenic in ClinVar. Only variants annotated as high or moderate impact on the gene product or having at least one splicing score predicting aberrant splicing were further analyzed. Compare File S3 sheet “hc-joint”.

Copy number variant (CNV) calling from panel data was performed using CNVkit [13] version 0.9.6. The parameters “target-avg-size” was set to 50 and “antitarget-avg-size” to 200.000 to optimize settings for the smaller panel design. The cohort was divided by the sequencing machine (MiSeq vs. HiSeq) and both sub-cohorts were randomly split into two equal sized groups which were used as control cohorts for each other. Resulting per sample CNV calls were annotated with their RefSeq based gene content and aggregated into a cohort list for filtering. Compare File S3 sheet “CNVkit”.

Variant evaluation and confirmation

Small variants (SNV/indel) were evaluated for their biological plausibility, examined for quality using the IGV browser and classified according to the five-tier variant classification system recommended by the American College of Medical Genetics and Genomics (ACMG) [14]. For carriers of a (likely) pathogenic variant in *CEP290*, we performed Sanger sequencing to exclude the deep intronic founder variant NM_025114.:c.2991+1655A>G (rs281865192; primers 5'-CATGGGAGTCACAGGGTAGG-3' and 5'-TGATGTTTAACGTTATCATTTTCCC-3'.

CNVs were visualized with the "scatter" and "heatmap" functions in CNVkit, They were then inspected in the IGV browser to compare their coverage profile with other samples, check the variant allele frequencies (VAF) at variant sites and search for break-point informative split reads. In the sample from individual "Ind_739404" we could identify split-reads supporting the heterozygous *COL4A5* deletion chrX:g.107731844_107920385del and confirmed the variant with exact breakpoints using allele specific PCR and Sanger sequencing (5'-AATTTGTTGCCTGTCTTTTGC-3' and 5'-TGCAGAATAAAACCCACACAAC-3'). The deletion ("Ind_958149") and duplication ("Ind_207310") affecting the *HNF1B* locus were confirmed using the MLPA kit P241 (MRC Holland, Amsterdam, Netherlands).

Analysis of the *MUC1-VNTR* region

We analyzed the typical cytosine duplication ("dupC") located at variable positions in the VNTR between exons 2 and 3 of *MUC1* with an established SNaPshot minisequencing protocol for all archived samples selected for panel sequencing[15]. Additionally, we had designed the panel to include capture probes targeting the *MUC1*-VNTR and included three *MUC1*-dupC positive controls in panel sequencing to enable bioinformatic analysis of this region. We used adVNTR[16] version 1.3.3 (https://github.com/mehrdadbakhtiari/adVNTR/) with custom settings "frameshift" mode and "vntr_id 25561" to identify indels in this complex genomic region.

Comparison with published screening data in CKD

We compared our analysis to the currently largest published sequencing study in CKD[17] by directly downloading all variants from this study from ClinVar (SCV000809114 to SCV000809473) as submitted by the authors using a custom R language script. Such downloaded HGVS nomenclature was converted to VCF format using the batch function in VariantValidator (https://variantvalidator.org). We then annotated the resulting VCF file with the pipeline described above for our cohort. Additionally, we annotated whether the respective variant could be detected by our panel using the panel design browser extensible data (BED) file. To harmonize the ACMG classification for our cohort and the Groopman cohort we used

the two automated AMCG classification tools integrated in VarSome (https://varsome.com) “ACMG Implementation” and VarSeq v2.2.3 “Sample ACMG Classifier” (Golden Helix, Inc., Bozeman, MT, USA; www.goldenhelix.com) for both variant sets with standard settings. We aggregated multiple (likely) pathogenic variants, as predicted by the ACMG classifiers, per individual in the Groopman cohort and performed 10.000 simulations drawing our final cohort sample size (n=271) from the Groopman cohort. In each simulation we counted how many individuals could be diagnosed by our panel or by exome and how many individuals would have a (likely) pathogenic variant in *COL4A5*. The results of this simulation were then compared with our diagnostic yield using only variants automatically classified as (likely) pathogenic and excluding CNVs and mitochondrial variants. Results were visualized using scatter and violin plots and empirical p-values were calculated by computing how many simulations had a higher or equal yield fraction or collagen IV variant fraction, respectively.

Statistical analyses and plotting

All data regarding cohort, panel content and identified variants were aggregated into Excel (Microsoft Corporation, Redmond, USA) files and are attached as supplementary to this article. These data were imported, analyzed and plotted using R language version 4.1.0 with RStudio IDE version 1.4.1717 (RStudio Inc., Boston, MA, USA). Libraries "broom", "cowplot", "DiagrammeR", "DiagrammeRsvg", "fs", "fuzzyjoin", "ggrepel", "readxl", "rsvg", "tidyverse" and "UpSetR". Inkscape 1.1 (https://inkscape.org/) was used to adjust Figure 1 and Figure 2 for parts which could not be directly composed in R. Schematic linear gene plots with variant positions represented as lollipops scaled to the variant’s CADD score[18] were created in R as described previously[19].

The two sided Wilcoxon signed-rank test as implemented in R was used to compare pairwise differences between groups (except for the p-values in the simulation estimated by sampling or when the question could be modeled as a Bernoulli experiment where we used the binomial test).

SUPPLEMENTARY RESULTS

Additional variants of unknown significance in 9.6%

Further 26 variants of unknown significance (VUS; ACMG class 3) in nine genes (*COL4A3*, *COL4A4*, *COL4A5*, *DNAJB11*, *GATM*, *HNF1B*, *MYH9*, *PARN*, *REN*) were identified in 26 (9.6%) individuals (Table S1). As the VUS category has the largest probability span from >10% to <90% chance of being pathogenic, we calculated the odds and p-values of pathogenicity using our manual classification criteria and recently published Bayesian framework.[20] Seven missense variants in seven genes (*COL4A3*, *COL4A4*, *COL4A5*, *DNAJB11*, *GATM*, *MYH9*, *REN*) had an odds-ratio of pathogenicity >9 and a probability of pathogenicity between 0.50 - 0.68. These "hot" class 3 variants could reach the likely pathogenic class with an additional moderate strength criteria like location in an established functional domain (PM1) or if another missense change at the same amino acid residue would be reported as pathogenic (PM5). With automated ACMG classifiers 3/7 (42.9%) of these "hot" VUS would be classified as likely pathogenic by Varsome and all seven (100%) as "VUS/Weak Pathogenic" by VarSeq. Overall, this indicates that further research and stringent deposition of diagnostic variants into public databases could further improve variant classification and thus increase diagnostic yield.

Nephronophthisis carrier status is not enriched

Interestingly, we identified 11 carriers (11/271 ~ 4.1%) for (likely) pathogenic variants in six nephronophthisis associated genes (4x *NPHP3*, 2x *CEP290*, 2x *IQCB1*, 1x *ANKS6*, 1x *TTC21B*, 1x *ZNF423*), yet not a single diagnostic case with a second (including VUS) variant *in trans* (Table S2). Thus, nephronophthisis does not appear to play a role in this adult cohort. We asked whether the observed carrier frequency in our cohort represents an enrichment, which could point to either missed deep intronic/ regulatory variants or to nephronophthisis carrier status being a risk factor for CKD. We thus downloaded all variants from the gnomAD database for the 17 nephronophthisis genes in our target design, classified them using the VarSeq classifier and used the allele frequencies for (likely) pathogenic variants detectable by our design to calculate the probability of being a variant carrier in at least one of these genes (~ 3.5%). As nine variants in our cohort (9/271 ~ 3.3%) would be automatically classified as (likely) pathogenic, the results indicate no enrichment and refutes our initial hypothesis. Knowing this high background carrier probability, reporting of heterozygous carrier status in individuals without a clear clinical suspicion of nephronophthisis should carefully be considered to not cause diagnostic uncertainty in individuals and clinicians.

Comprehensive *MUC1*-VNTR analysis identifies no *MUC1*-dupC

We performed diagnostic grade SNaPshot mini-sequencing[15] for the typical *MUC1*-dupC variant in the VNTR in all 271 archived DNA samples from the final cohort. For 225 individuals (83.8%) we obtained reliable results but did not identify a *MUC1*-dupC positive case. In 14 samples (5.2%) the results obtained could not reliably be evaluated and in the remaining 32 samples (11.8%) SNaPshot sequencing was not possible, likely due to low DNA quality. Due to the GCKD study design we could not re-contact the individuals/ clinicians to obtain new samples.

We had designed the panel target to directly cover the *MUC1*-VNTR with capture probes and included three known *MUC1*-dupC positive DNA samples from individuals previously diagnosed in our institute.[15,21] Using the adVNTR software, we could confirm the *MUC1*-dupC event ("I22_2_G_LEN1") in the three positive controls, but did not find this typical duplication or any other high confidence sequence variant in the VNTR, potentially leading to a similar aberrant protein product, in any of the 271 cohort samples.

Thus, combining SNaPshot mini-sequencing and bioinformatic analysis, no ADTKD-*MUC1* case (0/271) could be identified in this cohort of mostly sporadic kidney diseases (Figure 2G). Compare also File S3[22] sheets "SNaPshot" and "adVNTR" for complete per sample results.

SUPPLEMENTARY FIGURES

Figure S1 | Diagnostic yield and COL4 gene fraction simulations using automated variant classification

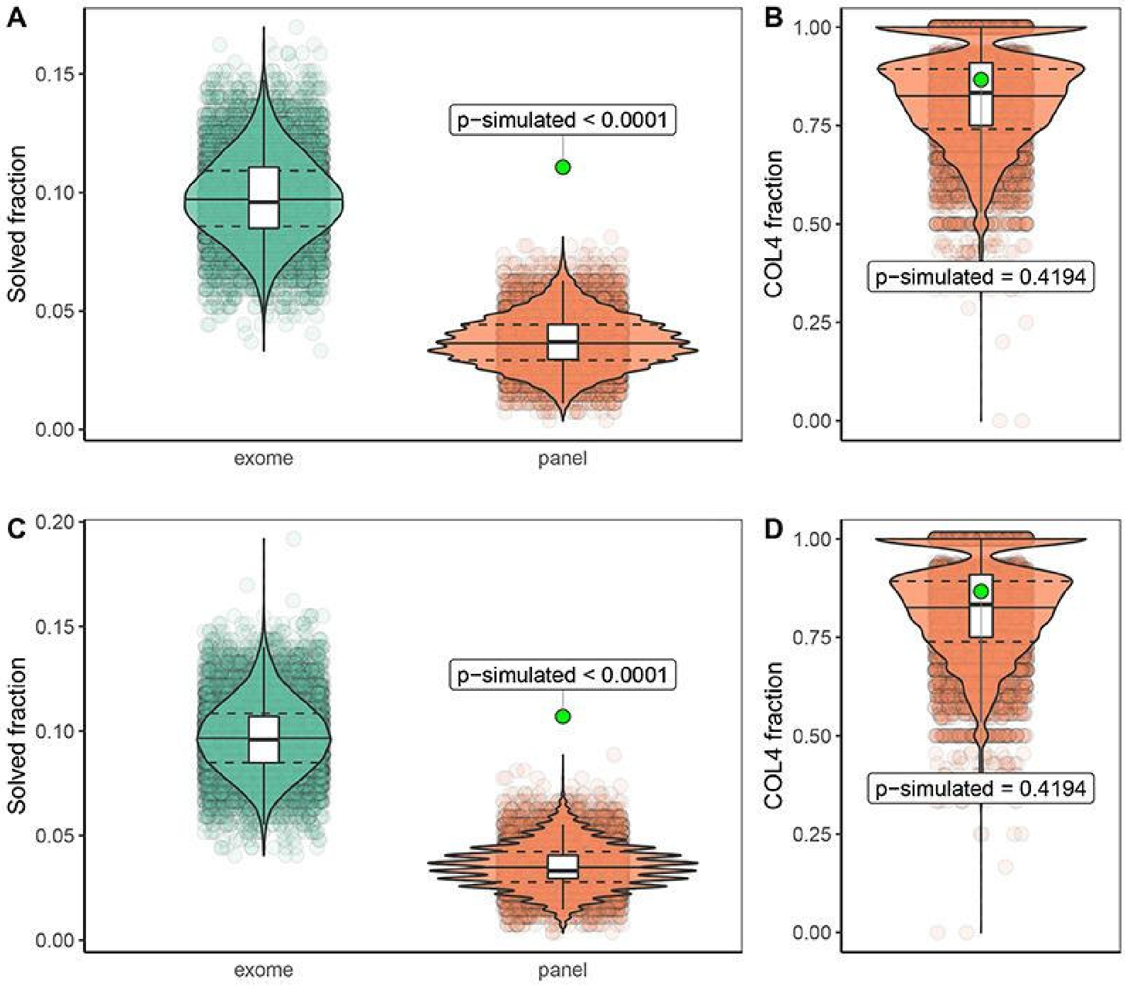

Results as in main Figure 1 but using automated variant classifications for both our and the Groopman cohort variants. **(A)** and **(B)** automated ACMG classification using Varsome. **(C)** and **(D)** automated ACMG classification using VarSeq.

Figure S2 | Albumin-Creatinine Ratio (ACR) by IgA nephropathy (IgAN) status and genetic diagnosis group

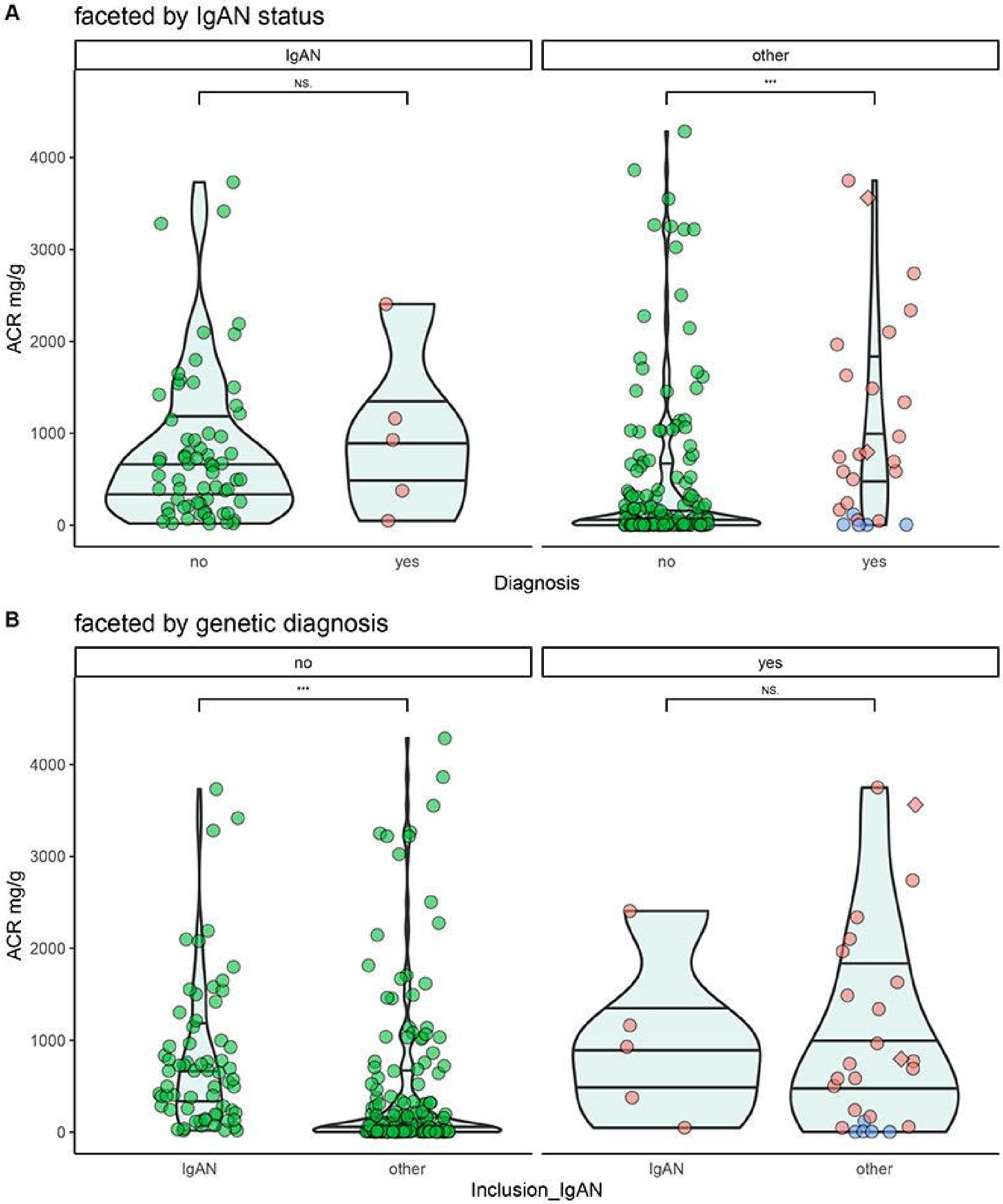

Data on ACR in the cohort from the right panel of Figure 3C faceted by **(A)** IgAN status (left clinical group with IgAN, right other groups) and **(B)** genetic diagnosis group (left no genetic diagnostic variant identified, left with (likely) pathogenic variant). Colored as in Figure 3C. Results indicate that individuals with clinical IgAN have significantly higher ACR, but this does not drive the significantly higher ACR observed in the individuals with a diagnostic variant. This might further support that the clinical classification of IgAN in these individuals with a diagnostic variant is not the primary cause of nephropathy.

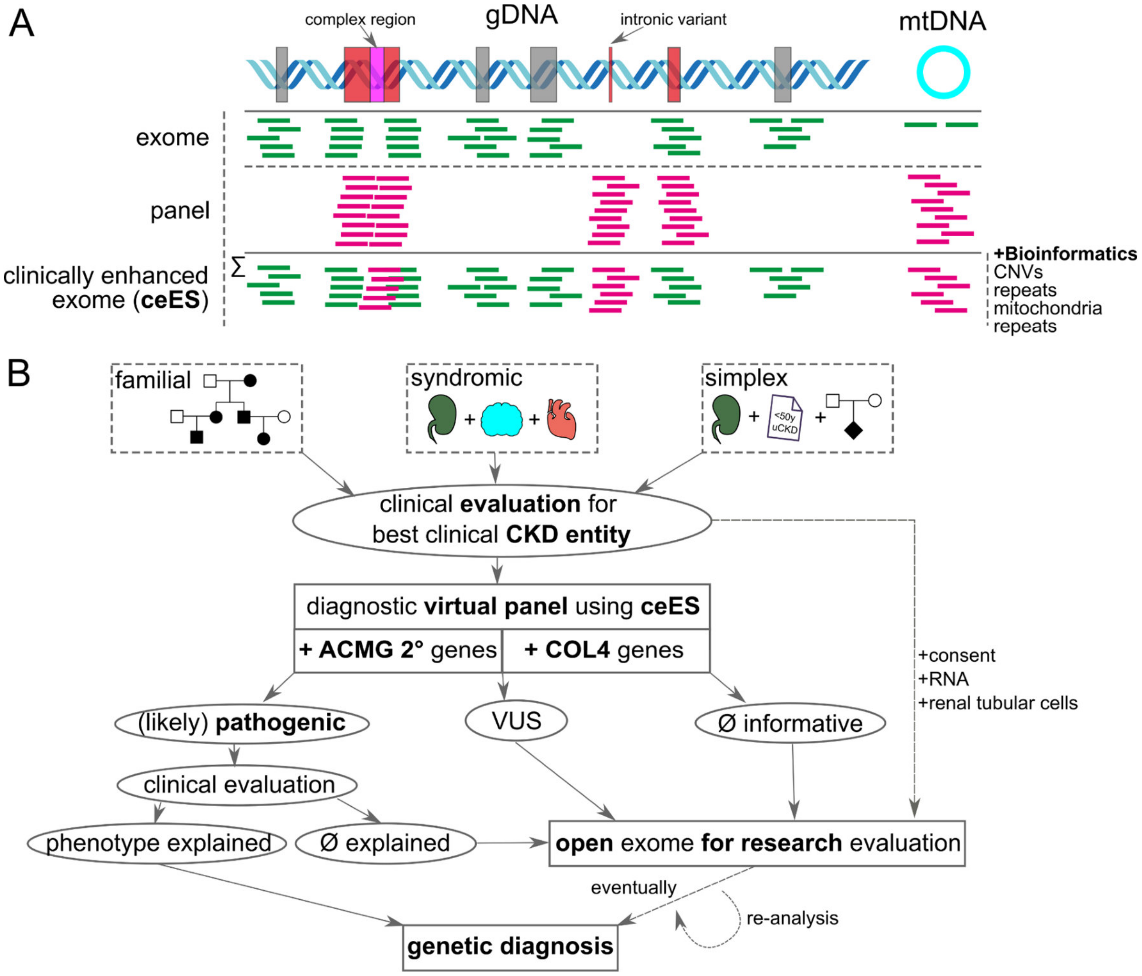

Figure S3 | Proposed clinically enhanced exome design and evaluation workflow
(A) Schematic figure explaining short read sequencing based panel and exome sequencing (ES) and their respective advantages. ES covers all coding exons but has gaps in complex regions (like the *MUC1*-VNTR or *PKD1* duplicated exons), may miss clinically relevant intronic variants and has low coverage for mtDNA. Custom panels in contrast can be designed to have high coverage of these regions but would need to be iteratively re-designed and re-sequenced for each possible disease entity. An ES target design enhanced through expert knowledge (several companies nowadays offer adding custom capture probes) allows adapting the design to the respective diagnostic needs (ceES). **(B)** Our proposed workflow to select individuals for genetic diagnosis is based on positive family history, syndromic disease (e.g. multiple organ systems affected) and isolated simplex cases without secondary cause of CKD younger than 50 years. Genetic diagnostics should be based on clinically selected virtual panels and include ACMG recommended secondary findings and *COL4A3*, *COL4A4* and *COL4A5* genes. Depending on the outcome and whether eventual variants explain the phenotype of the individual the ceES data should be opened to research analysis enhanced by possible RNA analyses and functional tests using e.g. renal tubular cells to finally reach a genetic diagnosis.

SUPPLEMENTARY TABLES

Gene	Variant	Type	ACMG criteria	Individual	Previous Clinical diagnosis	Kidney biopsy
COL4A3	c.685C>T, p.(Arg229Trp)	SNV	class 3 (p=0.50)	Ind_581621	nephrosclerosis	no
	c.1052A>C, p.(Gln351Pro)	SNV	class 3 (p=0.19)	Ind_190910	nephrosclerosis	no
	c.4421T>C, p.(Leu1474Pro)	SNV	class 3 (p=0.19)	Ind_501279	chronic glomerulonephritis	no
	c.4523A>G, p.(Asn1508Ser)	SNV	class 3 (p=0.19)	Ind_805187	unknown	no
	c.897C>T, p.(=)	SNV	class 3 (p=0.10)	Ind_313427	IgA nephropathy	yes
	c.4782C>T, p.(=)	SNV	class 3 (p=0.10)	Ind_485984	nephrosclerosis	yes
MYH9	c.979A>T, p.(Arg327Trp)	SNV	class 3 (p=0.68)	Ind_220163	others	no
	c.490+47G>A, p.?	SNV	class 3 (p=0.32)	Ind_200834	nephrosclerosis	yes
	c.2041G>A, p.(Gly681Ser)	SNV	class 3 (p=0.19)	Ind_399997	nephrosclerosis	yes
	c.3340T>C, p.(Ser1114Pro)	SNV	class 3 (p=0.19)	Ind_279807	nephrosclerosis	yes
	c.3340T>C, p.(Ser1114Pro)	SNV	class 3 (p=0.19)	Ind_702034	unknown	no
	c.5128A>G, p.(Ile1710Val)	SNV	class 3 (p=0.10)	Ind_819147	chronic glomerulonephritis	yes
COL4A4	c.4963G>A, p.(Val1655Met)	SNV	class 3 (p=0.68)	Ind_527531	others	no
	c.5045G>A, p.(Arg1682Gln)	SNV	class 3 (p=0.32)	Ind_257933	hereditary disorders	no
	c.1770A>T, p.(=)	SNV	class 3 (p=0.19)	Ind_791075	nephrosclerosis	no
	c.3398-3C>T, p.?	SNV	class 3 (p=0.10)	Ind_753616	nephrosclerosis	yes
PARN	c.1319-16T>A, p.?	SNV	class 3 (p=0.10)	Ind_737791	unknown	no
	c.1330C>T, p.(Arg444Cys)	SNV	class 3 (p=0.10)	Ind_554434	IgA nephropathy	yes

Gene	Variant	Type	ACMG criteria	Individual	Previous Clinical diagnosis	Kidney biopsy
	c.1501G>T, p.(Ala501Ser)	SNV	class 3 (p=0.10)	Ind_958149	unknown	no
DNAJB11	c.973G>C, p.(Asp325His)	SNV	class 3 (p=0.50)	Ind_180606	nephrosclerosis	no
	c.895C>G, p.(Leu299Val)	SNV	class 3 (p=0.10)	Ind_102360	unknown	no
HNF1B	c.1127C>T, p.(Thr376Ile)	SNV	class 3 (p=0.19)	Ind_501279	chronic glomerulonephritis	no
	c.1006C>G, p.(His336Asp)	SNV	class 3 (p=0.10)	Ind_533929	hereditary disorders	no
COL4A5	c.3095T>C, p.(Met1032Thr)	SNV	class 3 (p=0.68)	Ind_890866	unknown	no
GATM	c.875A>G, p.(His292Arg)	SNV	class 3 (p=0.50)	Ind_550368	interstitial nephritis	no
REN	c.699G>T, p.(Glu233Asp)	SNV	class 3 (p=0.50)	Ind_579540	IgA nephropathy	no

Table S1 | Additional variants of unknown significance

List of all individuals who had a VUS identified and their diagnostic group/s and whether they had a renal biopsy. Please compare Supplementary Notes for the calculation of the Bayesian p-values and File S3 for detailed criteria applied in manual ACMG variant classification.

Gene	Variant	Type	ACMG criteria	Individual	Previous Clinical diagnosis	Kidney biopsy
NPHP3	c.1381G>T, p.(Glu461*)	SNV	class 5 (p=0.99)	Ind_600000	IgA nephropathy	yes
	c.2694-2_2694-1del, p.0?	indel	class 5 (p=0.97)	Ind_581621	nephrosclerosis	no
	c.2694-2_2694-1del, p.0?	indel	class 5 (p=0.97)	Ind_834192	IgA nephropathy	yes
	c.1629-2A>G, p.0?	SNV	class 4 (p=0.90)	Ind_440439	nephrosclerosis; IgA nephropathy	yes
CEP290	c.5493del, p.(Ala1832Profs*19)	indel	class 5 (p=0.99)	Ind_613519	IgA nephropathy	yes
	c.292_293insA, p.(Leu98Hisfs*18)	indel	class 5 (p=0.99)	Ind_903214	IgA nephropathy	yes
IQCB1	c.1518_1519del, p.(His506Glnfs*13)	indel	class 5 (p=0.97)	Ind_181070	IgA nephropathy	yes
	c.1518_1519del, p.(His506Glnfs*13)	indel	class 5 (p=0.97)	Ind_245000	IgA nephropathy; chronic glomerulonephritis	no
ANKS6	c.130_157del, p.(Glu44Argfs*72)	indel	class 4 (p=0.90)	Ind_999093	nephrosclerosis	yes
TTC21B	c.1715C>A, p.(Ser572*)	SNV	class 4 (p=0.90)	Ind_753169	nephrosclerosis	no
ZNF423	c.2738C>T, p.(Pro913Leu)	SNV	class 5 (p=0.97)	Ind_890866	unknown	no

Table S2 | Nephronophthisis carrier variants

List of all individuals in which a heterozygous (likely) pathogenic variant in 17 nephronophthisis genes was identified together with their diagnostic group/s and whether they had a renal biopsy. Please compare Supplementary Notes for the calculation of the Bayesian p-values and File S3 for detailed criteria applied in manual ACMG variant classification.

WEB RESOURCES

gnomAD browser: http://gnomad.broadinstitute.org/

ClinVar: https://www.ncbi.nlm.nih.gov/clinvar/

VariantValidator: https://variantvalidator.org

adVNTR: https://github.com/mehrdadbakhtiari/adVNTR/

RNAfold: http://rna.tbi.univie.ac.at/cgi-bin/RNAWebSuite/RNAfold.cgi

MITOMAP: https://www.mitomap.org/MITOMAP

ABBREVIATIONS

ADTKD:autosomal dominant tubulointerstitial kidney disease

CKD: chronic kidney disease

COL4: Collagen-4 genes associated with Alport syndrome (*COL4A5*, *COL4A4* and *COL4A3*)

CNV: copy number variant

DNA: deoxyribonucleic acid

ES: exome sequencing

GCKD: German Chronic Kidney Disease

indel: insertion/ deletion variant

MITKD: mitochondrially inherited tubulointerstitial kidney diseases

NPHP: nephronophthisis

SNV: single nucleotide variant

SUPPLEMENTARY REFERENCES

1. Eckardt KU, Barthlein B, Baid-Agrawal S, et al. The German Chronic Kidney Disease (GCKD) study: design and methods. *Nephrol Dial Transplant*. 2012;27(4):1454-1460. doi:10.1093/ndt/gfr456
2. Titze S, Schmid M, Köttgen A, et al. Disease burden and risk profile in referred patients with moderate chronic kidney disease: composition of the German Chronic Kidney Disease (GCKD) cohort. *Nephrol Dial Transplant Off Publ Eur Dial Transpl Assoc - Eur Ren Assoc*. 2015;30(3):441-451. doi:10.1093/ndt/gfu294
3. Li H. Aligning sequence reads, clone sequences and assembly contigs with BWA-MEM. *ArXiv13033997 Q-Bio*. Published online May 26, 2013. Accessed July 11, 2021. http://arxiv.org/abs/1303.3997
4. McKenna A, Hanna M, Banks E, et al. The Genome Analysis Toolkit: A MapReduce framework for analyzing next-generation DNA sequencing data. *Genome Res*. 2010;20(9):1297-1303. doi:10.1101/gr.107524.110
5. Poplin R, Ruano-Rubio V, DePristo MA, et al. *Scaling Accurate Genetic Variant Discovery to Tens of Thousands of Samples*. Genomics; 2017. doi:10.1101/201178
6. Cingolani P, Platts A, Wang LL, et al. A program for annotating and predicting the effects of single nucleotide polymorphisms, SnpEff: SNPs in the genome of Drosophila melanogaster strain w1118; iso-2; iso-3. *Fly (Austin)*. 2012;6(2):80-92. doi:10.4161/fly.19695
7. Cingolani P, Patel VM, Coon M, et al. Using Drosophila melanogaster as a Model for Genotoxic Chemical Mutational Studies with a New Program, SnpSift. *Front Genet*. 2012;3. doi:10.3389/fgene.2012.00035
8. Liu X, Li C, Mou C, Dong Y, Tu Y. dbNSFP v4: a comprehensive database of transcript-specific functional predictions and annotations for human nonsynonymous and splice-site SNVs. *Genome Med*. 2020;12(1):103. doi:10.1186/s13073-020-00803-9
9. Xiong HY, Alipanahi B, Lee LJ, et al. The human splicing code reveals new insights into the genetic determinants of disease. *Science*. 2015;347(6218):1254806-1254806. doi:10.1126/science.1254806
10. Jian X, Boerwinkle E, Liu X. In silico prediction of splice-altering single nucleotide variants in the human genome. *Nucleic Acids Res*. 2014;42(22):13534-13544. doi:10.1093/nar/gku1206
11. Landrum MJ, Lee JM, Benson M, et al. ClinVar: improving access to variant interpretations and supporting evidence. *Nucleic Acids Res*. 2018;46(D1):D1062-D1067. doi:10.1093/nar/gkx1153
12. Stenson PD, Ball EV, Mort M, et al. Human Gene Mutation Database (HGMD ®): 2003 update: HGMD 2003 UPDATE. *Hum Mutat*. 2003;21(6):577-581. doi:10.1002/humu.10212
13. Talevich E, Shain AH, Botton T, Bastian BC. CNVkit: Genome-Wide Copy Number Detection and Visualization from Targeted DNA Sequencing. *PLOS Comput Biol*. 2016;12(4):e1004873. doi:10.1371/journal.pcbi.1004873
14. Richards S, Aziz N, Bale S, et al. Standards and guidelines for the interpretation of sequence variants: a joint consensus recommendation of the American College of Medical Genetics and Genomics and the Association for Molecular Pathology. *Genet Med Off J Am Coll Med Genet*. 2015;17(5):405-424. doi:10.1038/gim.2015.30
15. Ekici AB, Hackenbeck T, Morinière V, et al. Renal fibrosis is the common feature of autosomal dominant tubulointerstitial kidney diseases caused by mutations in mucin 1 or uromodulin. *Kidney Int*. 2014;86(3):589-599. doi:10.1038/ki.2014.72
16. Bakhtiari M, Park J, Ding YC, et al. Variable number tandem repeats mediate the expression of proximal genes. *Nat Commun*. 2021;12(1):2075. doi:10.1038/s41467-021-22206-z
17. Groopman EE, Marasa M, Cameron-Christie S, et al. Diagnostic Utility of Exome Sequencing for Kidney Disease. *N Engl J Med*. 2019;380(2):142-151. doi:10.1056/NEJMoa1806891

18. Rentzsch P, Witten D, Cooper GM, Shendure J, Kircher M. CADD: predicting the deleteriousness of variants throughout the human genome. *Nucleic Acids Res*. 2019;47(D1):D886-D894. doi:10.1093/nar/gky1016
19. Hebebrand M, Hüffmeier U, Trollmann R, et al. The mutational and phenotypic spectrum of TUBA1A-associated tubulinopathy. *Orphanet J Rare Dis*. 2019;14(1):38. doi:10.1186/s13023-019-1020-x
20. Tavtigian SV, Greenblatt MS, Harrison SM, et al. Modeling the ACMG/AMP variant classification guidelines as a Bayesian classification framework. *Genet Med Off J Am Coll Med Genet*. 2018;20(9):1054-1060. doi:10.1038/gim.2017.210
21. Wenzel A, Altmueller J, Ekici AB, et al. Single molecule real time sequencing in ADTKD-MUC1 allows complete assembly of the VNTR and exact positioning of causative mutations. *Sci Rep*. 2018;8(1):4170. doi:10.1038/s41598-018-22428-0
22. Popp, Bernt. Data files for manuscript "Prevalence of hereditary tubulointerstitial kidney diseases in the German Chronic Kidney Disease study." Published online September 19, 2021. doi:10.5281/ZENODO.5516388
